# Polyphenol Loaded W_1_/O/W_2_ Emulsions Stabilized with Lesser Mealworm (*Alphitobius diaperinus*) Protein Concentrate Produced by Membrane Emulsification: Stability under Simulated Storage, Process, and Digestion Conditions

**DOI:** 10.3390/foods10122997

**Published:** 2021-12-04

**Authors:** Junjing Wang, Aurélie Ballon, Karin Schroën, Sílvia de Lamo-Castellví, Montserrat Ferrando, Carme Güell

**Affiliations:** 1 Departament d’Enginyeria Química, Universitat Rovira i Virgili, Avda. Països Catalans 26, 43007 Tarragona, Spain; junjing.wang@urv.cat (J.W.); aurelie.ballon@urv.cat (A.B.); silvia.delamo@urv.cat (S.d.L.-C.); montse.ferrando@urv.cat (M.F.); 2 Laboratory of Food Process Engineering, Wageningen University, Bornse Weilanden 9, 6708 WG Wageningen, The Netherlands; karin.schroen@wur.nl

**Keywords:** insect protein, lesser mealworm, polyphenol encapsulation, multiple emulsions, membrane emulsification, emulsion stability

## Abstract

Water-in-oil-in-water (W_1_/O/W_2_) emulsions are complex delivery systems for polyphenols amongst other bio-actives. To stabilize the oil–water interphase, dairy proteins are commonly employed, which are ideally replaced by other, more sustainable sources, such as insect proteins. In this study, lesser mealworm (*Alphitobius diaperinus*) protein concentrate (LMPC) is assessed and compared to whey protein (WPI) and pea protein (PPI), to stabilize W_1_/O/W_2_ emulsions and encapsulate a commercial polyphenol. The results show that LMPC is able to stabilize W_1_/O/W_2_ emulsions comparably to whey protein and pea protein when using a low-energy membrane emulsification system. The final droplet size (d_4,3_) is 7.4 μm and encapsulation efficiency is between 72 and 74%, regardless of the protein used. Under acidic conditions, the LMPC shows a similar performance to whey protein and outperforms pea protein. Under alkaline conditions, the three proteins perform similarly, while the LMPC-stabilized emulsions are less able to withstand osmotic pressure differences. The LMPC stabilized emulsions are also more prone to droplet coalescence after a freeze–thaw cycle than the WPI-stabilized ones, but they are the most stable when exposed to the highest temperatures tested (90 °C). The results show LMPC’s ability to stabilize multiple emulsions and encapsulate a polyphenol, which opens the door for application in foods.

## 1. Introduction

Multiple emulsion-based delivery systems, especially water-in-oil-in-water (W_1_/O/W_2_) double emulsions, can be applied to tune the bioactive profile of foods, pharma products, and cosmetics, as they can encapsulate, protect, and release bioactive lipids (such as vitamin E) and water-soluble compounds such as vitamins B and C, flavorings, polyphenols, and probiotics [1,2,3]. Polyphenols are well-known, highly effective antioxidants that possess various health benefits, such as the prevention of cancer, inflammation, diabetes, and cardiovascular diseases [4,5]. They exist in a wide range of plants in nature, and are a well-known target for by-product or food-waste valorization, such as grape seeds [6], spent coffee grounds [7], carob pulp [8] and olive leaves [9]. Due to their sensitivity to light, heat, oxidation, and certain pH values, the encapsulation of polyphenols has been carried out using several technologies. Amongst them, water-in-oil-in-water (W_1_/O/W_2_) emulsions are a promising strategy based on their high encapsulation efficiency, chemical stability and increased bio-accessibility upon controlled release [4,10,11,12]. The incorporation of polyphenols encapsulated in W_1_/O/W_2_ emulsions into several food matrices, for instance, yoghurt [10,13], salad dressing [9], and meat products [14], have been recently reported in the literature.

During the formation of emulsions, the emulsifier plays an important role in both decreasing the interfacial tension and preventing coalescence of the droplets; therefore, the selection of a suitable emulsifier is a key step in the formulation of a stable emulsion system. W_1_/O/W_2_ emulsions commonly use lipophilic emulsifiers to stabilize the W_1_/O primary emulsion, such as polyglycerol polyricinoleate (PGPR), Span 80, and lecithin [3]. To stabilize W_1_/O emulsion in the W_2_ phase, in general, natural food-grade ingredients such as dairy proteins, whey, and casein from milk, are widely used because of their amphiphilic structure. From the perspective of green and sustainable development, it is of great significance to replace proteins from dairy sources with feasible alternatives from a sustainable source that can alleviate global warming. Thus, plant and insect proteins are promising alternatives that have attracted attention due to their techno-functional properties [15].

Plant proteins, such as protein isolated from peas (*Pisum sativum* L., PPI), are nutritious, gluten-free, non-genetically modified, and present low allergenicity [16]. PPI has been characterized as having techno-functional properties, and some emulsifying properties to stabilize O/W emulsions and Pickering emulsions, either solely or as PPI-polysaccharides conjugates [17,18,19,20]. The incorporation of plant proteins in the formulation of W_1_/O/W_2_ emulsions is little reported. Xu et al. [21] evaluated the stability of pigment-encapsulated W_1_/O/W_2_ emulsions stabilized by soy protein isolate under various temperatures and salt concentrations, which demonstrated great heat stability and a tolerance to <5 mM CaCl_2_, and Tamnak et al. [22] reported that W_1_/O/W_2_ emulsions stabilized by pectin-PPI conjugate resulted in a higher emulsion stability and zeta potential, smaller droplet size and better encapsulation properties than the emulsions stabilized with native pectin and Tween 80.

Insect proteins have drawn attention in recent years as a sustainable alternative to more classic animal proteins in several areas of the world, mainly those where insects are not habitually consumed. The Food and Agricultural Organization (FAO) promotes the consumption of insects due to their great nutritional value, lower greenhouse gas emissions during rearing, and a short lifecycle, with a great potential economic benefit [23], while challenges remain in the implementation of insects in food and feed [24,25]. In Europe, the first safety assessment on the use of dried yellow mealworm as novel food was reported in 2021, with encouraging results [26]. Edible insects have high protein content on a dry-weight basis, e.g., 44.8–50.1% in yellow mealworm (*Tenebrio molitor*), 42.0–45.8% in cricket (*Acheta domesticus*), 62.4–67.2% in grasshoppers (*Oedalius asiaticus*, *Angaracris rhodopa*, *Chorthippus dubius* and *C. fallax*), and 57.6% in lesser mealworm (*Alphitobius diaperinus*) [27,28].

Investigations on techno-functional properties including solubility, water- and oil-binding capacity, foaming capacity, surface hydrophobicity, gelling properties, coagulation properties, and the emulsifying ability of various insect powders, and their protein extracts have already shown promising outcomes [29,30,31,32,33,34,35]. In addition, enzymatic hydrolysis of the protein extracts could efficiently enhance their functionalities and obtain bioactive peptides with antioxidative, antihypertensive, antidiabetic, and antimicrobial properties [36,37,38,39,40,41,42]. Regarding studies in emulsions stabilized with insect proteins, Wang et al. [43] reported a superior performance of black soldier fly (*Hermetia illucens*) protein concentrate on emulsifying a high fraction (40 wt%) of lemon oil compared to whey protein isolate (WPI), and Gould and Wolf [44] found that sunflower oil emulsions stabilized with mealworm protein displayed smaller droplet size and a lower protein concentration was required compared to WPI; in addition, the produced emulsion also showed great stability under wide environmental stresses (temperature at −20 °C and 60–90 °C, pH 3–8 and ionic strength 80–330 mM). These results show the potential use of insect proteins to stabilize emulsions and are a good starting point to broaden their use to more complex systems, such as W_1_/O/W_2_ emulsions.

The objective of this work is to study the ability of lesser mealworm protein concentrate (LMPC) to stabilize W_1_/O/W_2_ emulsions designed to encapsulate and protect a commercial procyanidin-rich extract. The performance of the insect protein will be compared with a conventional dairy protein (whey protein) and a protein from another sustainable source (pea protein). The effect of several environmental stresses such as temperature, pH, and salt concentration on the emulsion stability will be assessed for LMPC and compared with the other two selected proteins. Special attention is paid to protein–polyphenol interactions. To the best of our knowledge, this is the first attempt to assess the use of an insect protein to stabilize W_1_/O/W_2_ emulsions, providing relevant results regarding its feasibility and potential applications in food, pharmaceuticals, and cosmetics.

## 2. Materials and Methods

### 2.1. Materials

The composition of double emulsions is listed in Table 1. Vitaflavan (DRT, Dax Cedex, France) is a red–violet-colored commercial white grape seed extract, with a reported total polyphenol content above 96% and antioxidant activity (ORAC) of 19,000 mmol TEQ g^−1^, which will be referred to hereafter as procyanidin-rich extract. 10 wt% procyanidin-rich extract solution was vacuum filtered through 11 μm pores (grade 1 filter paper, Whatman, Buckinghamshire, UK) before use. Polyglycerol polyricinoleate (PGPR, ref-4120 Palsgaard, Juelsminde, Denmark) was used as a lipophilic emulsifier dissolved in commercial sunflower oil (Borges S.A., Tarragona, Spain). Proteins investigated in this study are whey protein isolate (WPI, BiPRO, lot no. JE 034-7-440-6, Davisco Foods International. Inc., Eden Prairie, MN, USA) with a reported protein content of 98.1% on a dry basis, lesser mealworm protein concentrate (LMPC) was extracted from insect powder BUFFALO’S (Kreca Ento-Food BV, Wageningen, the Netherlands) at lab scale, and pea protein isolate (PPI, Roquette, NUTRALYS, s85F, Lestrem, France) had a reported purity of 80–90%. To produce LMPC, 2-methyl tetrahydrofuran (2-MeTHF, EMPLURA, Darmstadt, Germany) was used in pre-defatting, sodium hydroxide pellet (CHEM-LAB, Zedelgem, Belgium) and hydrochloric acid (37–38% HCl, J.T. Baker, Griesheim, Germany) were used for extraction.

A total of 2 wt% WPI solution was prepared one day before by dissolving WPI powder in 5 mM phosphate buffer pH 7 prepared with di-sodium hydrogen phosphate dihydrogen, (Scharlau, Spain) and sodium phosphate monobasic monohydrate (ACROS, Spain) under magnetic stirring for 2 h at 400 rpm and kept in the fridge overnight for complete hydration. LMPC and PPI solutions were prepared similarly to WPI using buffer, and pH adjustment to 7.0 every 30 min using 4 M NaOH or 1 N HCl. After two hours, the solutions were put in the fridge overnight. LMPC and PPI concentrations were quantified before use with the Pierce^TM^ BCA protein assay kit (Thermoscientific, Rockford, IL, USA) and concentrations were expressed as bovine serum albumin equivalent value (BSAE%, *w*/*w*). The concentration of WPI and the BSAE% of LMPC and PPI was obtained by dilution in buffer and the addition of sodium chloride (NaCl, Panreac, Barcelona, Spain), and antimicrobial agent sodium azide (NaN_3_, Sigma-Aldrich, Saint Louis, MO, USA), as indicated in Table 1. LMPC and PPI protein concentration is shown in % for simplicity reasons. Sodium carbonate (Panreac, Barcelona, Spain), gallic acid monohydrate (Panreac, Barcelona, Spain) and Folin-Ciocalteau’s reagent (Panreac, Barcelona, Spain) were used for total polyphenol content (TPC) quantification.

In the emulsification module, glass micro-beads with a size of 38 μm (Microspheres-nanospheres, New York, NY, USA) were placed on top of a nickel sieve (Stork Verco, Erbeek, the Netherlands). Sodium hydroxide and ethanol (96%, Scharlab, Sentmenat, Spain) were applied to clean nickel sieve and silica beads, respectively.

### 2.2. Preparation of Lesser Mealworm Protein Concentrate (LMPC)

Defatting of the original lesser mealworm powder was carried out using the organic solvent 2-MeTHF. In brief, 50 g of lesser mealworm whole-fraction powder was mixed with 250 mL of 2-MeTHF in a covered beaker and stirred magnetically (RCT ST, IKA, Staufen, Germany) at 300 rpm for 1 h. Then, decantation of the solvent layer was carried out after complete phase separation. The process was repeated 3 times in total by adding 250 mL of 2-MeTHF each time. The remaining solvent in the powder was evaporated in the fume hood over 3 days. Protein extraction was conducted based on the literature [44] with slight modifications. In brief, 30 g dried defatted powder and 150 mL of 0.25 M NaOH solution were stirred at 400 rpm for 1 h at 40 °C. The mixture was centrifuged (Meditronic 7000599, J.P. SELECTA, Barcelona, Spain) for 15 min at 3358× *g*. The supernatant was separated, and its pH was adjusted to 4.0–4.5 with HCl to precipitate protein; the remaining pellet was subsequently used for protein extraction, repeating the process two more times. The precipitated protein from the 3 extractions was combined and centrifuged (15 min, 2343× *g*); then, it was freeze-dried (LYOQUEST-85 PLUS, Telstar, Barcelona, Spain) for 24 h at 0.2 mbar vacuum and plate temperature at 20 **°C**. The collected freeze-dried protein powders were blended and stored in a desiccator with a water activity of 0.075.

### 2.3. Osmolality of W_1_ and W_2_

Osmolality of water phases was measured using vapor pressure osmometer (K-7000, KNAUER, Berlin, Germany) at 39 + 2 °C calibrated by 400 mOsmol/kg NaCl solution. To balance the relatively high osmolality of the 10% procyanidin-rich extract solution (W_1_), NaCl was added to the protein solutions (W_2_) as shown in Table 1. Results are shown as mean ± standard deviation (*n* = 5).

### 2.4. W_1_/O/W_2_ Emulsions Production

#### 2.4.1. Coarse W_1_/O/W_2_ Emulsion

The method of producing W_1_/O/W_2_ emulsions followed the one reported by Wang et al. [8]. In brief, the primary emulsion (W_1_/O) was generated by the addition of W_1_ phase solution into sunflower oil with 6% PGPR under rotor-stator homogenization (Ultra Turrax T18 digital, IKA, Staufen, Germany) for 5 min at 11,000 rpm. Then, the primary emulsion was introduced into W_2_ phase, containing the dissolved protein, while being stirred at 1600 rpm for 5 min on a magnetic stirrer to produce coarse W_1_/O/W_2_ emulsions.

#### 2.4.2. Refinement of W_1_/O/W_2_ emulsions by Dynamic Membranes of Tunable Pore Size (DMTS)

**Figure 1:**
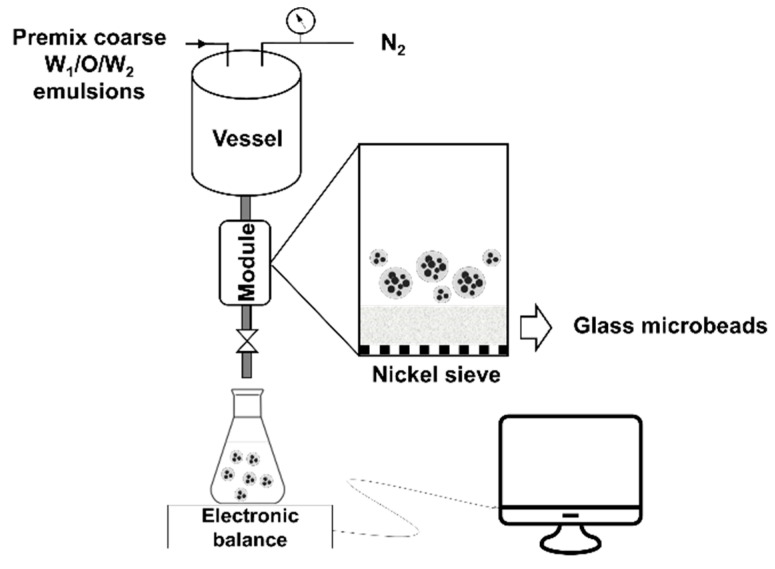
Schematic representation of experimental DMTS setup.

DMTS consists of a layer of glass microbeads placed on top of a nickel sieve (Figure 1). The nickel sieve had pores of 284.7 × 12.8 μm (length × width) with a thickness of 120 μm. Glass microbeads of 38 μm (0.44 g) were placed in the module on top of the nickel sieve, which resulted in 2 mm layer with an interstitial void diameter of ~22 μm, as calculated using the literature [43]. Coarse emulsions were placed in the vessel and immediately passed through the DMTS system pressurized to 500 kPa with N_2_; this process is called one emulsification cycle. Three emulsification cycles were conducted for W_1_/O/W_2_ emulsions stabilized with the three proteins. Refined emulsions were collected separately in a flask placed on an electronic balance to record the mass gain every second, from which the transmembrane flux can be calculated as:

(1) $$J_{DMTS}=\frac{\phi }{\rho_{e}\;A}$$

where ϕ is the mass flow rate, as acquired from the data recorded with the electronic balance; $\rho_{e}$ is the emulsion density; A is the effective surface area of the DMTS.

‘Blank’ emulsions were produced following an identical process, except that the W_1_ phase consisted of a 0.4 wt% NaCl solution. The purpose of preparing blank emulsions is to eliminate the impact of proteins on the total polyphenol content quantification.

The DMTS module was disassembled after use, and nickel sieves and glass microbeads were reused after cleaning and drying, following the cleaning protocol described earlier [8]. Duplication was carried out for each formulation.

### 2.5. Environmental Stress Test

Freshly produced W_1_/O/W_2_ emulsions were divided over several glass tubes that were tightly shut for further treatment at various environmental conditions. Samples were covered with aluminum foil to avoid light and stored at the required conditions, depending on the environmental stress test performed. The stability of the emulsions was followed using droplet size distribution, microstructure (microscopic images), visual appearance, zeta potential, and encapsulation efficiency.

#### 2.5.1. pH

The influence of pH on emulsion stability was examined by adjusting the pH of the outer water phase of W_1_/O/W_2_ emulsions to 1.5, 4.0, and 8.0 (pH deviations of 0.25) with 35–37% HCl or 1 M NaOH. Samples were analyzed after storing them for 1, 7, and 14 days at room temperature.

#### 2.5.2. Temperature

The influence of temperature on emulsion stability was studied by storing the samples at −20 °C (in the freezer) for 24 h, at 4 °C (fridge) and 25 °C (room temperature) for 14 days, as well as individually holding the samples at 37 and 65 °C for 30 min and at 90 °C for 5, 15, 30, and 60 min on a dry bath heating block (FB15101, Fisher Scientific, Hemel Hempstead, UK). The latter three temperatures were chosen to represent the situation in the body, and during processing. Frozen samples were analyzed after thawing at room temperature. Samples maintained at 4 and 25 °C were analyzed after 1, 3, 7, and 14 days of storage. Samples subjected to heat treatment at 37, 65, and 90 °C were kept at room temperature for 24 h before analysis.

#### 2.5.3. Osmotic Stress

The influence of osmotic imbalance on emulsion stability was assessed from a 10-fold water dilution of W_2_ fraction (leading to osmotic pressure П_W1_ > П_W2_) and addition of NaCl to W_2_ phase to create an extra 50 or 250 mM salt concentration (resulting in osmotic pressure П_W1_ < П_W2_). Samples were stored at room temperature and analyzed after 1, 7, and 14 days of storage.

### 2.6. Characterization of Emulsions

#### 2.6.1. Droplet Size Distribution

Droplet size distribution (d_4,3_ and span) of W_1_/O/W_2_ emulsions was measured after each emulsification cycle and during environmental stress tests by laser diffraction using Mastersizer 2000 (Malvern Instruments, Worcestershire, UK). 0.4 wt% NaCl water solution was used as the continuous phase in Mastersizer Hydro 2000 G accessory to disperse the emulsion at a similar aqueous phase osmotic pressure. The particle reflective index and the dispersant reflective index were set to 1.480 and 1.330, respectively.

#### 2.6.2. Zeta Potential

Zeta potential of freshly produced W_1_/O/W_2_ emulsions and during environmental stress tests were measured using dynamic light scattering (Zetasizer Nano-ZS, Malvern Instruments, Worcestershire, UK) in triplicate. The changes in zeta potential at altered pH values were also monitored over 14 days. The same values of reflective index of particle and dispersant were applied as indicated in Section 2.6.1. Samples were diluted 200 times by deionized water.

#### 2.6.3. Encapsulation Efficiency of Polyphenols

Polyphenol encapsulation efficiency was deduced following the previously described method [8]. Blank W_1_/O/W_2_ and W_1_/O/W_2_ emulsions containing procyanidin-rich extract were centrifuged (Biocen 22R, Orto Alresa, Madrid, Spain) for 10 min at 825× *g*. Then, the W_2_ phase was carefully taken by needle and syringe, and total polyphenol concentration was analyzed based on Folin–Ciocalteau colorimetric method in triplicate. In brief, 100 μL of diluted sample and 100 μL of Folin reagent were mixed with 2 mL of 75 gL^−1^ Na_2_CO_3_ solution and 2.8 mL of deionized water. After 1 h of incubation at room temperature in the dark, absorbance was measured at 750 nm by a UV-Vis spectrophotometer (Hach Lange DR5000, Hach Lange SLU, Barcelona, Spain). The concentration of polyphenol was calculated using a calibration curve with a known amount of gallic acid as standard and expressed as gram gallic acid equivalent per liter (gGAE L^−1^). The mass of polyphenols that remained encapsulated in W_1_ was expressed as polyphenol encapsulation efficiency (*EE*) by Equation (2) [45]:

(2) $$EE\left[\%\right]=\frac{m_{poly_{W_{1}}}^{0}-C_{poly_{W_{2}}}\left(m_{W_{1}}^{0}+m_{W_{2}}^{0}\right)}{m_{poly_{W_{1}}}^{0}-C_{poly_{W_{2}}}m_{W_{1}}^{0}}\times 100$$

where $m_{poly_{W_{1}}}^{0}$ is the initial polyphenol mass in the inner water phase (W_1_), $C_{poly_{W_{2}}}$ is the concentration of polyphenols in the outer water phase (W_2_), $m_{W_{1}}^{0}$ is the initial mass of the inner water phase, and $m_{W_{2}}^{0}$ is the initial mass of the outer water phase.

#### 2.6.4. Microstructure Analysis

Laser scanning confocal microscope (NIKON model TE2000-E, Amsterdam, The Netherlands) was used to observe the W_1_/O/W_2_ emulsion structure and estimated droplet sizes.

### 2.7. Interaction of Protein and Polyphenols

A total of 0.5 wt% WPI (0.4 wt% NaCl) and 0.5% LMPC (0.06% NaCl) in 5 mM phosphate buffer (pH 7) solutions were used to assess the interaction with the procyanidin-rich extract (dissolved in a pH 7 phosphate buffer with 0.02 wt% NaN_3_) at concentrations ranging from 0 to 0.3%. The mixture was stirred, and the pH was adjusted in the range of 2.5 to 7.5 by adding 0.1 M NaOH or 0.1 N HCl dropwise. The transmission of the solution was then measured by static multiple light-scattering using Turbiscan Lab Expert (Formulation, Toulouse, France). The mixture was also centrifuged for 10 min at 3000 rpm to observe sediment formation. Duplicates were carried out for each polyphenol concentration.

### 2.8. Statistical Analysis

Data were analyzed using R (ver. 4.04, R Development Core Team, Vienna, Austria). One-way analysis of variance (ANOVA) and Tukey test were used to evaluate the significance between different emulsion samples with a level of 0.05.

## 3. Results

### 3.1. Production of W_1_/O/W_2_ Emulsions Stabilized with LMPC, WPI, and PPI

#### 3.1.1. Particle Size Distribution and Transmembrane Flux

**Figure 2:**
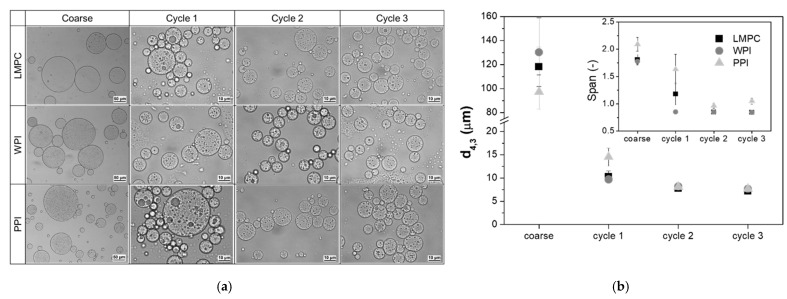
(**a**) Microscopic images (scale bars on coarse emulsion indicate 50 μm, and for remaining cycles 10 μm) and (**b**) d_4,3_ and span of W_1_/O/W_2_ emulsions stabilized with 1% LMPC (lesser mealworm protein concentrate), 1% WPI (whey protein isolate) and 1% PPI (pea protein isolate) as a function of emulsification cycle.

W_1_/O/W_2_ emulsions stabilized with three different emulsifiers, LMPC, WPI, and PPI, were produced following the procedure described in Section 2.4. The microstructure of these emulsions is presented in Figure 2a. It is clear from this figure that the W_1_/O/W_2_ emulsions were successfully formed with all emulsifiers. The overall variations in d_4,3_ and span after emulsification cycles (Figure 2b) followed the same trend for all the emulsions. Although coarse emulsions produced with different proteins resulted in differences in droplet size (d_4,3_ = 97.3–130.2 μm), the DMTS system successfully sharply refined them to 9.7 ± 2.4 μm after the first emulsification cycle, and then to 7.4 ± 0.2 μm after the third cycle, regardless of the protein used. The span of WPI-stabilized emulsions sharply decreased to 0.85 after the first cycle, while for LMPC and PPI emulsions the span reached this value after the second emulsification cycle. Therefore, the type of protein used to stabilize the oil-W_2_ interface did not considerably affect the final droplet size distribution of the W_1_/O/W_2_ emulsions after three emulsification cycles when using the DMTS system. The results show, as previously reported by other authors, that it is possible to reduce coarse emulsions with a droplet size 2–3.5 times bigger than the interstitial void diameter of the glass microbeads layer to a value smaller than the interstitial void diameter (less than 50%) [46]. Moreover, the droplet size distribution obtained using LMPC, WPI and PPI is similar to the one reported by Sahin et al. [47] for the production of W_1_/O/W_2_ emulsions stabilized with Tween 20 using the same emulsification technology with 30 μm glass microbeads. Overall, LMPC has shown a comparable potential to stabilize W_1_/O/W_2_ emulsions to WPI and PPI when using the low-energy membrane emulsification system.

An important parameter to scale up the emulsification process is the transmembrane flux. Table 2 presents the values obtained during the three emulsification cycles and shows that the values (which are all high, especially when compared to regular membrane emulsification) and evolution are very similar for all three proteins. The lowest flux corresponds to the first emulsification cycle, during which the highest droplet break-up occurs, and the energy is mostly invested in this process rather than in flowing the emulsion through the DMTS system. During the second and third cycles, there is a higher flux since almost no droplet break-up occurs (Figure 2b), and the pressure is mostly invested in flowing the emulsion through the system. The fluxes reported in the literature using the same system differ from 0.5 to 1200 m^3^m^−2^h^−1^ [48,49]. The differences in flux among the reported studies is expected, and not always comparable, as they are related to various factors such as the composition of emulsion, DMTS setup (glass microbeads size and amount, nickel sieve size) and applied pressure. Overall, the flux values obtained with the three proteins are high and in the range of industrial interest.

#### 3.1.2. Encapsulation Efficiency (EE)

In addition to the ability of the proteins to stabilize the newly formed interfaces during the emulsification cycles, leading to a narrow droplet size distribution (Section 3.1.1), it is also important to focus on their ability to retain the bioactive compound in the W_1_ phase during the emulsification process. It can be seen from Table 2 that all the emulsions have a similar EE, regardless of the protein used. For the coarse emulsions, the EEs could reach as high as 86.1–89.8%; this reduced to 74.0–76.2% after the first emulsification cycle, when major droplet break-up takes place, followed by a minor decrease (<2%) in the subsequent emulsification cycles. Emulsions stabilized with LMPC and WPI showed slightly higher values of EE than PPI for all the emulsification cycles. The evolution of EE obtained for the three proteins is similar to those reported for W_1_/O/W_2_ emulsions produced by membrane emulsifications using DMTS [8,49] and regular membrane emulsification [6,45,50].

### 3.2. Influence of Environmental Factors

The stability of emulsions formulated with proteins is generally affected by heat, pH, high ionic strength, and protease activity [51]. Since our W_1_/O/W_2_ emulsions may undergo different environmental conditions during processing, storage, and digestion, such as temperature, pH, and salt concentration, we studied changes in droplet size distribution, zeta potential, polyphenol release, and microstructure.

#### 3.2.1. pH

To simulate the acidity of various food substrates (such as acidic soft drinks and neutral nutritional beverages) and the ingestion process (in mouth, stomach, and intestine), emulsions were incubated at various pH values (1.5, 4.0, and 8.0) at ambient temperature for 14 days, and compared with emulsions at pH 6.5–7 (original pH).

**Figure 3:**
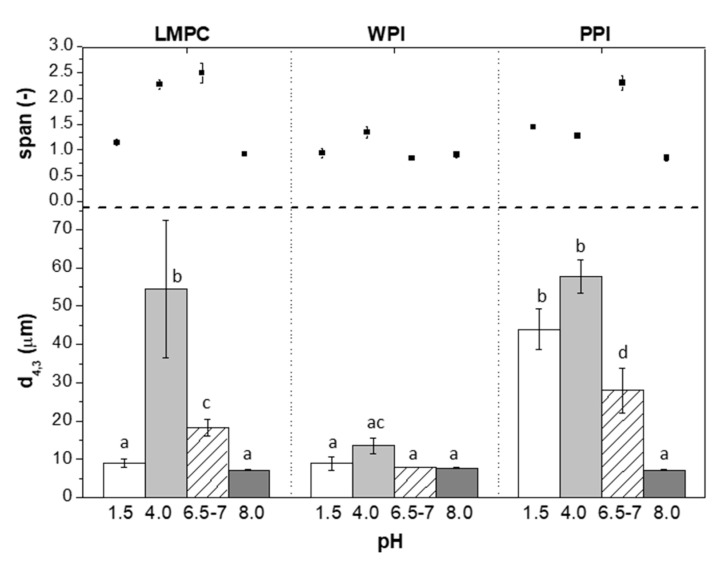
Droplet size distribution (d_4,3_ and span) of W_1_/O/W_2_ emulsions stabilized with LMPC, WPI and PPI after 14 days of incubation at pH 1.5, 4.0, 6.5–7, and 8.0. Different letters on top of the bar mean significant differences at *p* < 0.05.

**Figure 4:**
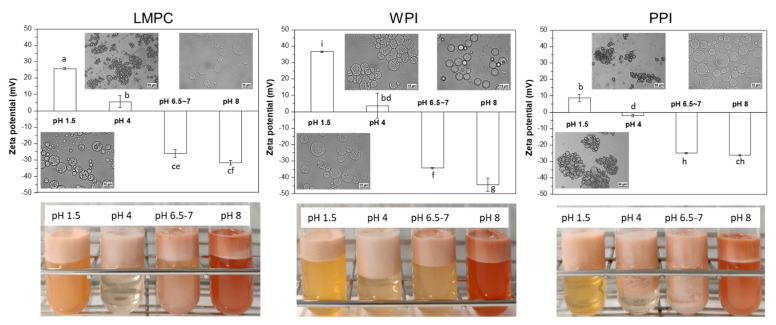
Effect of pH on zeta potential of W_1_/O/W_2_ emulsions stabilized with LMPC, WPI and PPI after 14 days of incubation, and visual appearance of the emulsions. Insets show microstructure of the emulsions following adjustment to pH 1.5, 4.0, 6.5–7, and 8.0. Different letters on top of the bar mean significant differences at *p* < 0.05.

**Figure A1:**
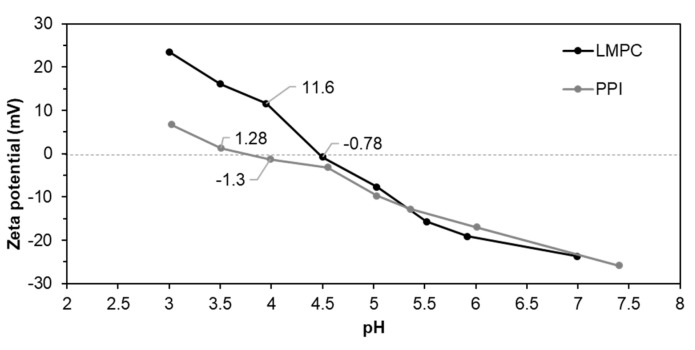
Zeta potential of LMPC (lesser mealworm protein concentrate) and PPI (pea protein isolate) solutions at pH range of 3.0–7.5.

Figure 3 shows the droplet size distribution of emulsions stabilized with LMPC, WPI, and PPI after 14 days of incubation at different pH values. At alkaline conditions (pH 8.0), all the emulsions show a similar droplet size distribution, irrespective of the emulsifier used, close to the initial value of pH 6.5–7 for LMPC and WPI and even lower for PPI. The negative surface charge (see Appendix A, Figure A1) was stronger at pH 8, which promotes droplet stabilization by enhanced electrostatic repulsion. Under acidic conditions (pH 1.5), emulsions stabilized with WPI or LMPC were stable, while they presented aggregation at pH 4.0, resulting in increased size (which is related to floc formation). In contrast, the emulsions stabilized with PPI showed significant aggregation at both pH 1.5 and 4.0 (inset images in Figure 4). The zeta potential, as presented in Figure 4, is indicative of emulsion stability, with values exceeding 20 mV, either plus or minus, leading to stable systems. This range coincides with the reported emulsion stability. Iso-electric point titrations of LMPC and PPI are shown in Figure A1, with clear differences at a low pH.

A similar pH stability of O/W emulsions stabilized with *T. molitor* protein extract was reported by the authors of [44]. Flocculation was observed at pH 4, and no significant changes at pH 2, 6, and 8, which is in line with our findings. Liang and Tang [52] observed that a solubilized PPI at pH 3 showed better emulsifying properties than those at a neutral or basic pH for the production of soy oil–water emulsions, suggesting that, under acidic conditions, pea protein forms a stronger and viscoelastic network. From the results in the present study, it can be concluded that PPI as we used it is not as efficient at stabilizing multiple emulsions as single emulsions at an acidic pH, while LMPC and WPI have a better performance.

To determine the total extent of polyphenol release, protein–polyphenol binding also needs to be taken into account. It has been reported that proteins and polyphenols can form aggregates by both non-covalent bonding, such as hydrogen bonding, hydrophobic bonding and van der Waals forces, and covalent binding interactions, which are affected by temperature, pH, salt concentration, and the presence of certain reagents [53,54,55,56,57]. From previous studies [6,8] it was clear that the interaction between the polyphenol and WPI in the W_2_ phase did not interfere with the polyphenol quantification method. A similar behavior was observed when analyzing EE in the emulsions stabilized with LMPC, WPI and PPI during the refining step with DMTS. In any of these cases, no increase in turbidity or the presence of a precipitate in W_2_ was noted, which led us to conclude that no protein–polyphenol binding took place.

**Figure 5:**
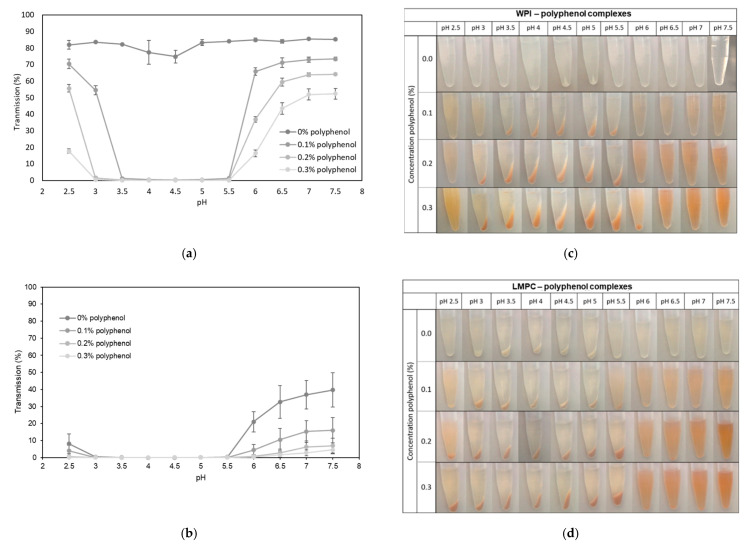
Transmission of (**a**) WPI and (**b**) LMPC solutions with polyphenol concentrations at 0, 0.1, 0.2, and 0.3% versus pH range of 2.5 to 7.5, with attached visual observation of sediments after centrifugation (**c**,**d**).

We first decided to investigate the effect of protein–polyphenol binding in aqueous solutions exposed to different pH levels, prior to exploring double emulsions. As Figure 5a shows, the formation of an insoluble protein–polyphenol complex was confirmed for WPI through the reduced transmission of WPI solution at a pH range of 3.5–5.5, leading to colored precipitate after centrifugation (Figure 5c). Upon increasing the polyphenol concentration, the transmission was further reduced, and the amount of visible colored precipitate increased. For LMPC (Figure 5b), the effect was even more pronounced, although it is notable that the protein also showed severe aggregation by itself in the pH range of 2.5–5.5. Comparing the transmission values of WPI and LMPC, in Figure 5a,b, respectively, we tentatively conclude that complex formation between the procyanidin-rich extract is more extensive and intensive for LMPC. Perhaps more importantly, it is clear from these results that the analysis of total polyphenol content in W_2_ could be compromised by the formation of protein–polyphenol complexes (especially within the pH range of 2.5–5.5), since it would only register polyphenols remaining in solution.

#### 3.2.2. Temperature

Next, the effect of temperature was investigated for conditions that would occur during the production, storage, and utilization, e.g., freezing, cooling, pasteurization, sterilization, and ingestion of food products. The influence of storage temperature (−20, 4 and 25 °C) and heating (37, 65 and 90 °C) on microstructure, droplet size distribution, and polyphenol release in W_1_/O/W_2_ emulsions was examined.

**Figure 6:**
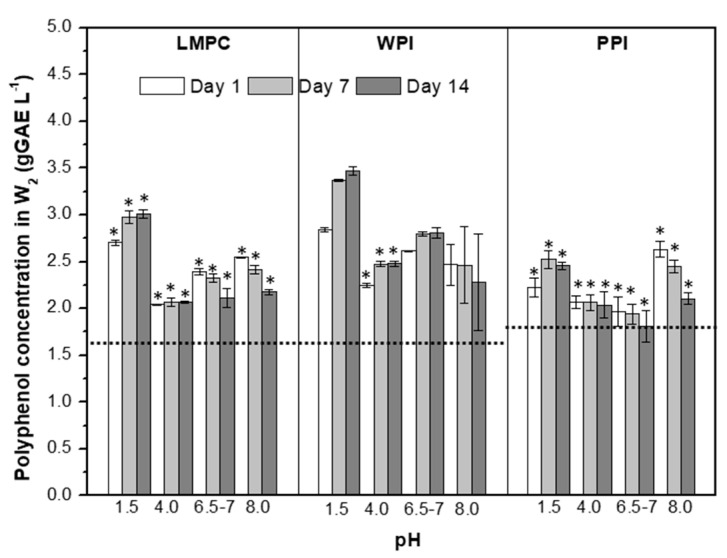
Polyphenol concentration in W_2_ of emulsions stabilized with LMPC, WPI and PPI during a quiescent storage at pH 1.5, 4.0, 6.5–7, and 8.0 over 14 days at room temperature. Asterisk labels above the bars point out the appearance of a precipitate during polyphenol analysis. Dashed lines indicate the value in freshly produced emulsions.

**Figure A2:**
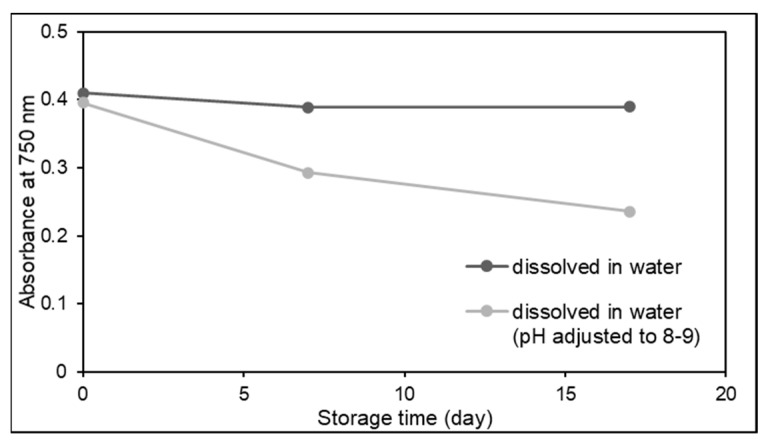
The variation of absorbance of polyphenols (Folin-Ciocalteau colorimetric method) from their solutions prepared in different media with storage time.

Polyphenol release can only be completely accounted for when no precipitate is formed, which was only the case at pH 1.5 and 8 for WPI. From Figure 6, it can be seen that, at pH 1.5, there is a significant release of polyphenols during the first 24 h, which increases during the first week of storage, reaching the highest value after 14 days. The comparative values at pH 8 seem to decrease over time, possibly as a result of degradation under alkaline conditions, as shown in the measurements presented in Appendix A, Figure A2, and mentioned elsewhere [58,59].

**Figure 7:**
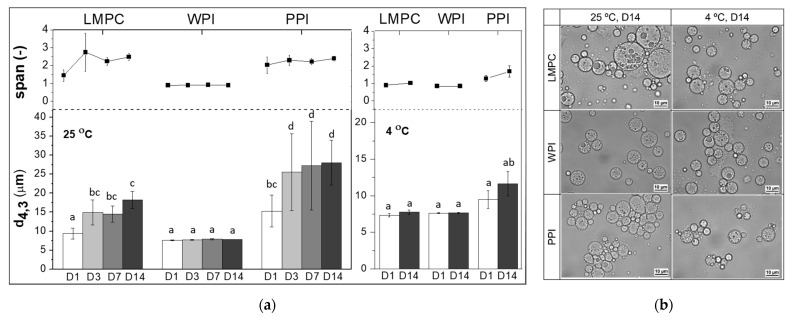
(**a**) Influence of storage temperature (25 and 4 °C) and storage time on droplet size distribution (d_4,3_ and span) of W_1_/O/W_2_ emulsions stabilized with LMPC, WPI, and PPI. D1, D3, D7, and D14 correspond to storage time of 1, 3, 7, and 14 days. (**b**) Microstructure of emulsions after 14 days storage at 25 and 4 °C. Different letters on top of the bar mean significant differences at *p* < 0.05.

The stability of the three emulsions in terms of droplet size distribution presented large differences when stored at room temperature (25 °C) and under refrigeration (4 °C) (Figure 7a), depending on the protein used to stabilize the O-W_2_ interphase. Emulsions stabilized with WPI did not show any changes in the droplet size distribution at 25 and 4 °C for two weeks, while the ones stabilized with LMPC or PPI showed an increase in size and span at 25 °C (also as a result of flocculation), and had better stability when stored at 4 °C. Compared to PPI, emulsions with LMPC are more stable in terms of droplet size distribution at both 25 and 4 °C. From the emulsion microstructure images (Figure 7b), the ‘increase in droplet size’ of LMPC and PPI-stabilized emulsions seem to be caused by droplet flocculation/aggregation rather than coalescence. PPI-stabilized emulsions started to flocculate immediately after production (Figure 2a), with flocs easily redispersed during the droplet size distribution analysis in the Mastersizer. These effects are in line with the results reported by Hinderink et al. [60,61] for low PPI concentration (<1%). All emulsions have considerable negative zeta potentials: PPI (−24.0 ± 3.7 mV), followed by LMPC (−29.5 ± 1.5 mV), and WPI (−37.9 ± 1.7 mV). This implies that all emulsions should have considerable charge stabilization with the emulsions, with the lowest charge being more susceptible to flocking.

**Figure 8:**
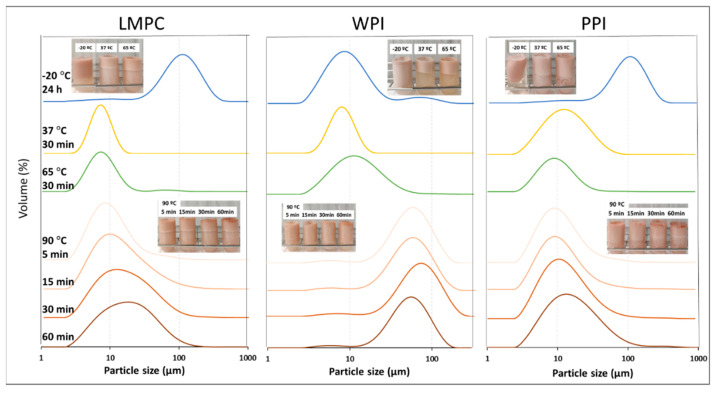
Droplet size distribution of W_1_/O/W_2_ emulsions stabilized with LMPC, WPI, and PPI after simulated heat-storage/-treatment conditions (― –20 °C for 24 h, ― 37 °C for 30 min, ― 65 °C for 30 min, and 90 °C for ― 5 min, ― 15 min, ― 30 min and ― 60 min), and visual appearance of the emulsions.

**Figure 9:**
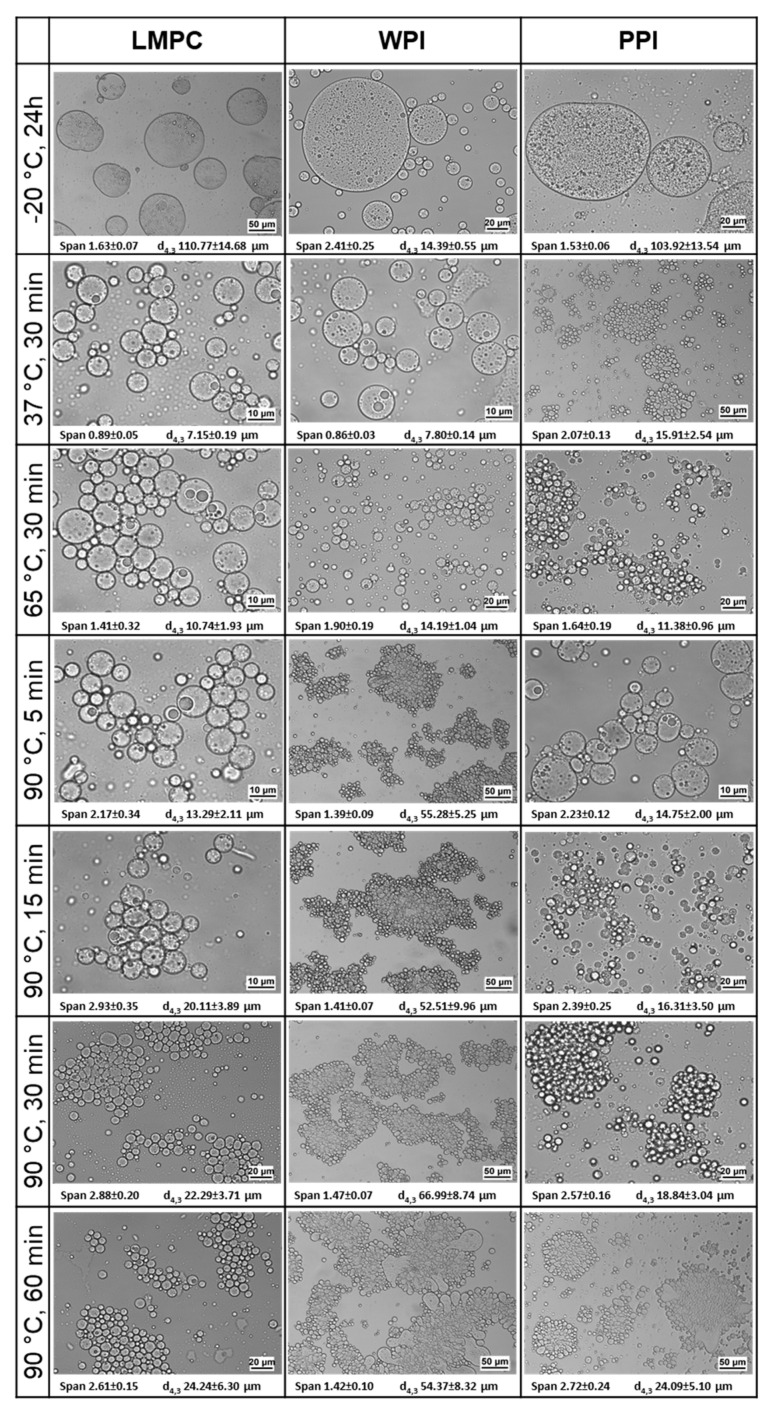
Microstructure of W_1_/O/W_2_ emulsions stabilized with LMPC, WPI, and PPI after simulated heat storage/treatment conditions (–20, 37, 65, and 90 °C).

W_1_/O/W_2_ emulsions were also tested under simulated freezing storage temperature (−20 °C) for 24 h and later analyzed at room temperature, i.e., one freeze–thaw process. From the results of droplet size distribution (Figure 8) and the microstructure (Figure 9), it is clear that all the samples were destabilized by coalescence and flocculation to a different extent, depending on the protein used in the emulsion formulation. Emulsions with WPI showed better freeze–thaw stability, maintaining a high-volume percentage of droplets with the initial droplet size and with a smaller population of larger droplets. LMPC and PPI emulsions showed an increase in the droplet size and increased coalescence, as displayed in the microscopic images (Figure 9). Mao et al. [62] reported a similar observation on the droplet size change on whey protein stabilized O/W emulsions after the first freeze–thaw cycle, where droplet coalescence intensified with the increase in the number of freeze–thaw cycles.

During the freezing process, oil droplets would concentrate in the unfrozen region and be densely packed, while the ice crystals that formed in the water phase could penetrate the oil globules and destroy the interfacial film, thus promoting droplet flocculation and coalescence during the thawing process [63]. Some studies on the freeze–thaw stability of O/W and Pickering emulsions found a correlation between the type and thickness of the stabilizing agent [62,64,65,66], as we found here. It is known that the thickness and viscoelasticity of the interfacial film layer determine the coalescence stability of a protein-stabilized emulsion [67], albeit not necessarily under cold conditions. β-lactoglobulin, as one of the main components of whey protein, provides interface viscoelastic characteristics due to a high 2D packing density and strong protein–protein interactions [68].

The W_1_/O/W_2_ emulsions produced in this study are more complex, since two interfaces (W_1_/O and O/W_2_) have to simultaneously remain stable; therefore, various mechanisms can result in destabilization during the freeze–thaw process [69]. From the microstructure observation in the micrographs (Figure 9), some degree of coalescence of inner water droplets can be assumed and coalescence of the droplets seems to have occurred, regardless of the protein used to stabilize the O/W_2_ interphase. Comparing the d_4,3_ for each emulsion after the freeze–thaw cycle, the W_1_/O/W_2_ emulsions stabilized with WPI show a mean value about of 14 μm, while, for LMPC and PPI the values, are 111 and 104 μm, respectively (Figure 9). Moreover, using LMPC and PPI leads to a higher droplet aggregation, as is evident from the clarified bottom aqueous phase (Figure 8). To improve the stability of LMPC emulsions at freezing conditions, the addition of a cryoprotectant is suggested to avoid protein denaturation, but this goes beyond the scope of the present study.

The emulsions were also exposed to temperatures relevant to digestion (37 °C), pasteurization (65 °C), and a more severe temperature treatment, during which protein denaturation takes place (90 °C). Before measuring droplet size distribution, the samples were gently mixed. Emulsions stabilized with WPI showed a decrease in stability with the increase in temperature (Figure 8), which can be linked to the denaturation of WPI. Droplet size distribution did not show any changes at 37 °C, but when the temperature increased to 65 and 90 °C (for 5 min), d_4,3_ increased to 14 and 55 μm, respectively. The denaturation temperature of whey proteins is around 65–85 °C [63]; therefore, emulsion destabilization was expected to take place. From the microstructure, it can be seen that most of the aggregates consisted of oil globules (W_1_/O) of a similar size to the freshly produced W_1_/O/W_2_ emulsions, accompanied by a small portion of coalesced droplets.

The emulsions stabilized with LMPC showed a similar stability to WPI emulsions at 37 and 65 °C, and seemed to less affected by heating at 90 °C than the ones with WPI. From the size distribution curve and the d_4,3_ values (Figure 8 and Figure 9) and microstructures (Figure 9) at 90 °C, it is clear that destabilization has taken place. Gould and Wolf [44] observed that 20% sunflower O/W emulsion stabilized by 0.44% *T. molitor* protein extract was stable during heat treatment at 60 and 70 °C, and the droplet aggregation commenced and strengthened when heated up to 80 and 90 °C, which is in line with our findings.

Emulsions stabilized with PPI presented a similar stability to LMPC during heating, even though, for PPI, increased droplet aggregation was observed at 37 °C, which further increased with temperature (Figure 9). As observed in the microscopic images (Figure 9), droplets of PPI-stabilized emulsions tended to form flocs or aggregates at 37 °C, which was not seen in emulsions stabilized with LMPC. We expect that the denaturation temperature for LMPC will be higher than that of WPI (65 °C) [70] and PPI (75–85 °C) [16], and this will translate into improved stability of the emulsions during short heat-treatments at 80–90 °C.

**Figure 10:**
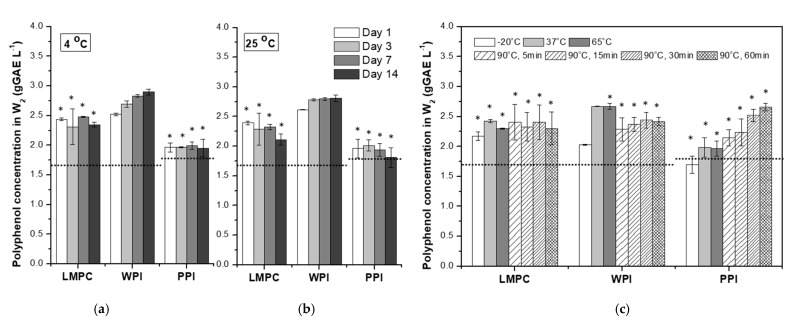
Polyphenol concentration in W_2_ of emulsions ($C_{poly_{W_{2}}})$ stabilized with LMPC, WPI and PPI during storage at 4 (**a**) and 25 °C (**b**) over 14 days, and (**c**) after being subjected to different temperature stresses: –20 °C (24 h), 37 °C (30 min), 65 °C (30 min) and 90 °C (5, 15, 30, and 60 min). Asterisk labels above the bars point to the appearance of a precipitate during polyphenol analysis. Dashed lines indicate the value for freshly produced emulsions.

Figure 10 shows the evolution of $C_{poly_{W_{2}}}$ for W_1_/O/W_2_ emulsions during the temperature stress tests. First, it is important to mention that the emulsion pH lies in the area (6.5–7), where the protein–polyphenol interaction is negligible for WPI and could be of significance depending on the polyphenol concentration of LMPC. The release of polyphenols to the W_2_ phase for emulsions stabilized with WPI showed a considerable increase after one day of storage at 4 and 25 °C, which could be due to the concentration gradient between W_1_ and W_2_. If all the polyphenol loaded into the W_1_ phase was released to the W_2_ phase, the concentration would be 6.2 g GAE L^−1^; considering this, it can be seen from Figure 10 that more than 50% of the loaded polyphenol is still encapsulated after 14 days at 4 and 25 °C for WPI emulsions. For the higher temperature tests (65 and 90 °C), the presence of a precipitate hinders direct polyphenol quantification, which is also the case for the LMPC and PPI emulsions (Figure 10). From the emulsion droplet size evolution during the different heat treatments (Figure 8), similar to the result obtained for WPI, we expect an overall retention of more than 50%.

#### 3.2.3. Osmotic Stress

The influence of osmotic pressure differences on the stability of W_1_/O/W_2_ emulsions was investigated through changes in droplet size distribution. The addition of salt and water to W_2_ led to an imbalance in osmotic pressure (П) between the two aqueous phases (W_1_ and W_2_), and the difference between W_2_ and W_1_ [Δ(П_W2_–П_W1_)] corresponded to 0.46 MPa, 2.42 MPa and −0.54 MPa for the addition of salt concentration of 50 and 250 mM and 10-fold water dilution, respectively.

**Figure 11:**
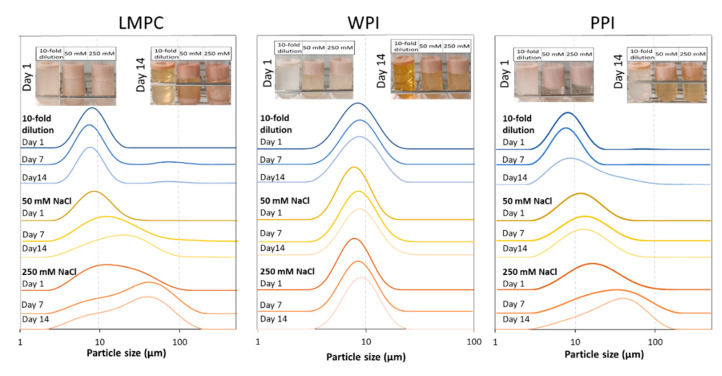
Droplet size distribution of W_1_/O/W_2_ emulsions stabilized with LMPC, WPI and PPI after addition of 50 mM NaCl (Δ(П_W2_–П_W1_) = 0.46 MPa), 250 mM NaCl (Δ(П_W2_–П_W1_) = 2.56 MPa) and 10-fold water dilution (Δ(П_W2_–П_W1_) = −0.54 MPa) of W_2_ over a storage period of 14 days; inset images show the visual appearance of the emulsions after 1 and 14 days of storage.

**Figure 12:**
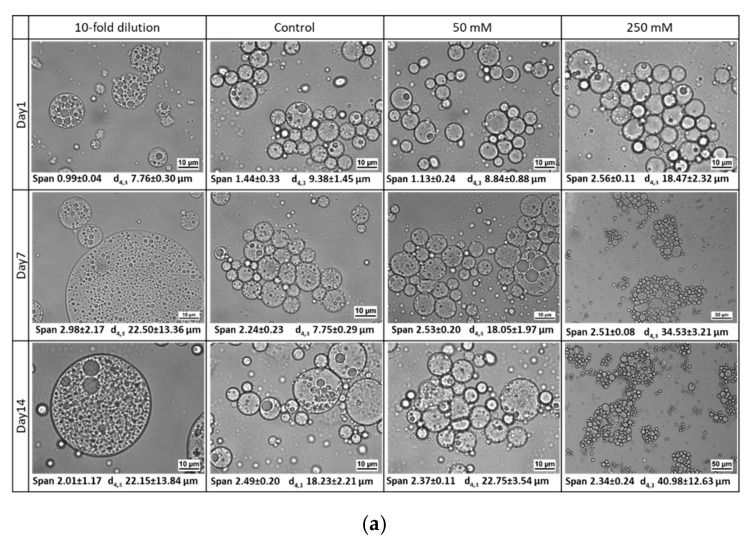

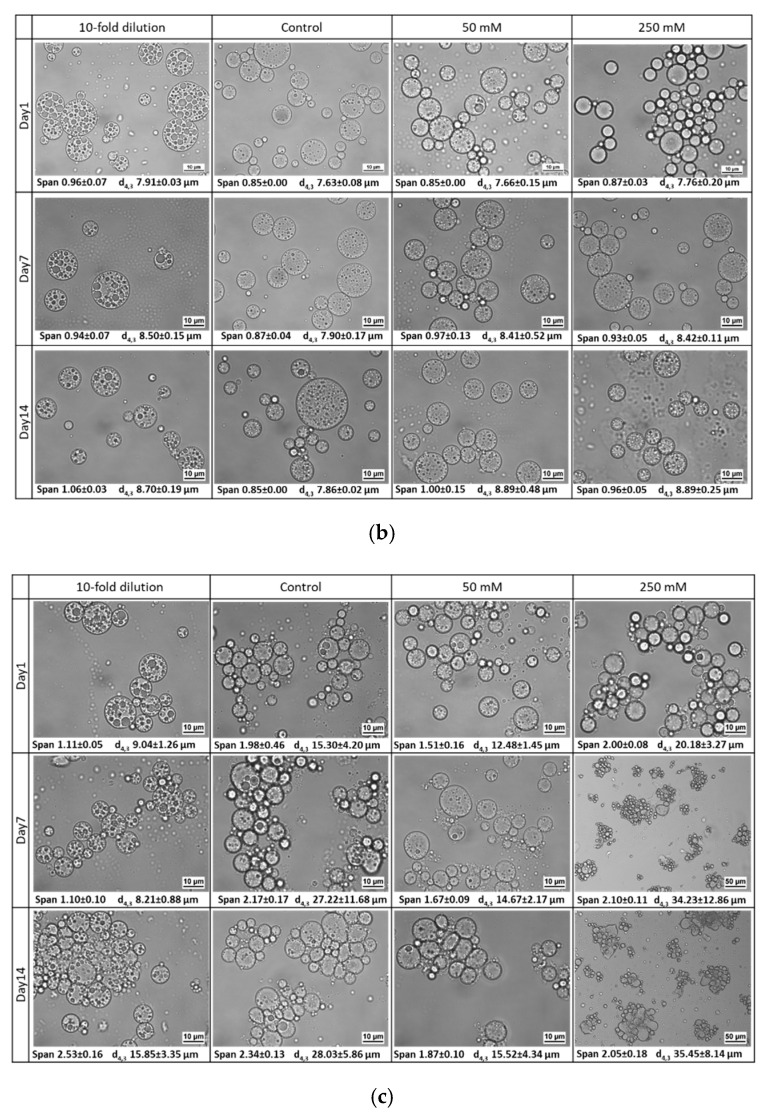
Microstructure of W_1_/O/W_2_ emulsions stabilized with LMPC (**a**), WPI (**b**) and PPI (**c**) after addition of 50 mM NaCl (Δ(П_W2_–П_W1_) = 0.46 MPa), 250 mM NaCl (Δ(П_W2_–П_W1_) = 2.56 MPa) and 10-fold of water dilution (Δ(П_W2_–П_W1_) = −0.54 MPa) of W_2_ during a storage period of 14 days.

As can be seen in Figure 11, W_1_/O/W_2_ emulsions stabilized with WPI maintained an approximately constant droplet size distribution during 14 days of storage, regardless of the changes in W_2_ phase. From the change in the color intensity of the W_2_ phase (bottom part of tube), it is clear that polyphenol release increases with storage time. For W_1_/O/W_2_ emulsions stabilized with LMPC, no significant change in droplet size distribution was observed for the emulsion with the diluted W_2_ phase during the first 7 days of storage. Some bigger droplets emerged after this, as seen from the microstructure images (Figure 12a). For the PPI-stabilized emulsions, dilution of the W_2_ phase changed the droplet size distribution, which is related to flocculation. The microstructure images (Figure 12) suggest a slight increase in the size of the inner water droplets, caused by the water efflux from W_2_ to W_1_ due to the imbalance in osmotic pressure.

Adding salt to any emulsion will influence charge effects that are responsible for emulsion stability [71,72,73]. In double emulsions, this can also induce water transfer between the two water phases. The addition of salt has an undoubted detrimental effect on the stability of W_1_/O/W_2_ emulsions with LMPC and PPI, where the internal water droplets most probably shrink over storage time and with the amount of salt added. If all the inner water is released to the outer phase, the resulting diameter would be 90% of the original emulsion droplet size, which cannot be distinguished by size measurement. There is a clear shift in droplet size toward bigger droplets/aggregates, while the polyphenol release is comparable to emulsions stabilized with WPI (visual observation). The most stable emulsions, under the examined range of salt concentration, were the ones produced with WPI, followed by LMPC and PPI. The tested salt concentrations represent a suitable range, as would be found in food products [74], and the encapsulation systems can withstand these salt concentrations. There are no previous studies in the literature on the stability of multiple emulsions stabilized with insect proteins. The only reference is a study on the stability of sunflower O/W emulsions stabilized with *T. molitor* protein extract, which, once produced, were subjected to a NaCl concentration of 330 Mm [44] and remained stable.

**Figure 13:**
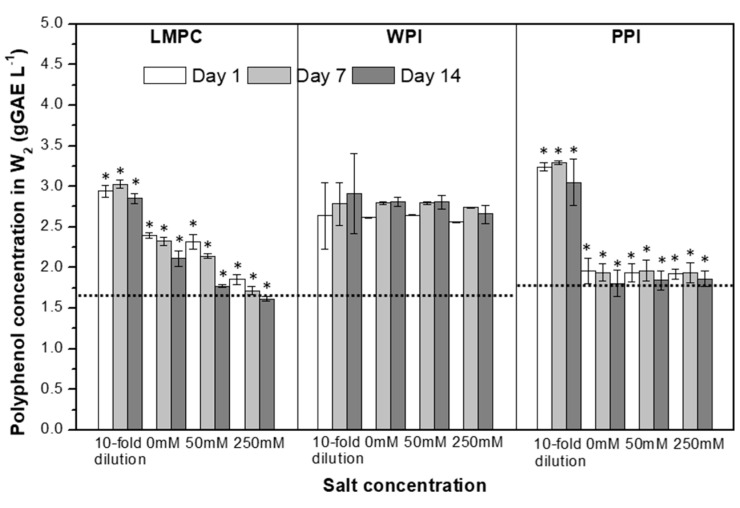
Polyphenol concentration in W_2_ of emulsions stabilized with LMPC, WPI and PPI during a quiescent storage of 14 days at room temperature. Emulsions were subjected to different conditions of 10-fold dilution by water (Δ(П_W2_–П_W1_) = −0.54 MPa), 0 (Δ(П_W2_-П_W1_) ≈ 0 MPa, with no extra addition of salt, kept at 25 °C), 50 mM NaCl (Δ(П_W2_–П_W1_) = 0.46 MPa) and 250 mM NaCl (Δ(П_W2_–П_W1_) = 2.56 MPa). Asterisk labeled above the bars points to the appearance of a precipitate during the polyphenol analysis. Dashed lines indicate the value in freshly produced emulsions.

To find out if water transport has any effects on the polyphenol release under the present experimental conditions, polyphenol concentration in W_2_ was measured after 1, 7 and 14 days of each osmotic stress test and plotted in Figure 13. For WPI-stabilized emulsions, it can be seen that the polyphenol concentration in W_2_ has a similar value (about 2.75 gGAE L^−1^) to the one obtained at 25 °C and a neutral pH and is not affected by the presence of salt (Figure 13); therefore, we can assume that more than 50% of the loaded polyphenols remain encapsulated under the tested osmotic stress conditions. For LMPC and PPI emulsions, the presence of a precipitate hampers the polyphenol analysis. Moreover, it has been established (results not shown) that the salt present in W_2_ enhances complex formation between LMPC and polyphenols (Figure 13). Contrary to the temperature stress tests, emulsion stability for LMPC and PPI was further compromised by the salt addition in W_2_, which could indicate that polyphenol release is higher than that for WPI emulsions.

## 4. Conclusions

This study has proven the feasibility of using lesser mealworm protein concentrate to stabilize multiple emulsions and encapsulate a procyanidin-rich extract. The emulsions showed a droplet size distribution and stability at 4 °C that is comparable to the emulsions produced with whey protein isolate. Moreover, the insect protein showed a better performance when stabilizing the multiple emulsions than a protein obtained from another sustainable source, such as pea protein. The interaction/complex formation between the LMPC and polyphenols is far more pronounced than that for WPI, which may be instrumental in improving the chemical stability of such systems.

For the stability under different environmental stresses (temperature, pH, and osmotic pressure imbalance), it was shown that all W_1_/O/W_2_ emulsions were prone to droplet coalescence after a freeze (−20 °C)–thaw cycle. At the highest temperatures tested (90 °C), the changes in droplet size distribution were less pronounced for W_1_/O/W_2_ emulsions stabilized with LMPC than those for WPI, pointing to a potential benefit of using this protein in emulsions that need to undergo heat treatment. Within the investigated pH range, LMWC-stabilized double emulsions behave comparably to their WPI-stabilized counterparts W_1_/O/W_2_ emulsions under acidic and alkaline conditions, and both outperform PPI. LMPC- and PPI-stabilized emulsions are less able to withstand osmotic pressure differences than WPI.

The results of this study show, for the first time, the use of an insect protein, LMPC, to stabilize multiple emulsions produced using a low-energy emulsification system to encapsulate a commercial polyphenol. This opens the door to future studies investigating the incorporation of insect proteins in complex food formulations.

## Figures and Tables

**Table 1:** Formulations of W_1_/O/W_2_ emulsions and osmotic properties of aqueous phases.

Phase	Fraction (%)	Composition	Osmolality (mOsmol/kg)	Calculated Osmotic Pressure (MPa)
W_1_	6	10 wt% procyanidin-rich extract	105.65 ± 4.97	0.56 ± 0.03
O	14	6 wt% PGPR in sunflower oil	--	--
W_2_	80	1 wt% WPI in phosphate buffer pH 7 (0.4 wt%, 0.02 wt% NaN_3_)	107.83 ± 1.07	0.57 ± 0.01
		1% LMPC in phosphate buffer pH 7 (0.06 wt% NaCl, 0.02 wt% NaN_3_)	110.03 ± 2.78	0.58 ± 0.01
		1% PPI in phosphate buffer pH 7 (0.25 wt% NaCl, 0.02 wt% NaN_3_)	104.93 ± 1.24	0.55 ± 0.01

**Table 2:** Transmembrane fluxes and EE (encapsulation efficiencies) of double emulsions stabilized with 1% LMPC (lesser mealworm protein isolate), 1% WPI (whey protein isolate), and 1% PPI (pea protein isolate) at each emulsification cycle.

	Flux (m^3^m^−2^h^−1^)			EE (%)		
	LMPC	WPI	PPI	LMPC	WPI	PPI
Coarse	--	--	--	87.3 ± 1.4	89.8 ± 0.1	86.1 ± 2.2
Cycle 1	110.0 ± 14.6	95.5 ± 2.2	137.9 ± 41.0	76.2 ± 0.3	75.0 ± 0.7	74.0 ± 2.3
Cycle 2	392.2 ± 23.0	377.1 ± 6.1	389.5 ± 48.3	75.2 ± 0.8	74.4 ± 1.2	72.8 ± 1.7
Cycle 3	387.5 ± 19.0	402.8 ± 3.4	389.1 ± 37.7	74.6 ± 0.1	74.2 ± 0.9	72.4 ± 1.4
